# Case Report: Treatment of fulminant paraquat poisoning in a 32-week pregnant patient: a case report exploring the potential role of estrogen

**DOI:** 10.3389/fphar.2026.1864030

**Published:** 2026-09-24

**Authors:** Li Wang, Mei Yu, Chunrong Chen, Xiaobing Li

**Affiliations:** 1 Department of Occupational Disease and Poisoning Medicine, The First Affiliated Hospital of Chongqing Medical and Pharmaceutical College, Chongqing, China; 2 Department of Neurology and Diabetes, Jiangbei District Hospital of Traditional Chinese Medicine, Chongqing, China; 3 Science and Technology Industry Development Center, Chongqing Medical and Pharmaceutical College, Chongqing, China

**Keywords:** acute lung injury, estrogen, gestational toxicology, maternal-fetal exchange, oxidative stress, paraquat poisoning, pregnancy, pulmonary fibrosis

## Abstract

Paraquat (PQ) poisoning is a life-threatening condition characterized by severe toxicity and the absence of a specific antidote. Prognosis is strongly correlated with the ingested dose, with high-dose exposure frequently resulting in multi-organ failure and fatal outcomes. This case report presents the clinical management of a 32-week pregnant patient with fulminant PQ poisoning, a scenario typically associated with near-certain mortality. Remarkably, the patient survived, supported by a markedly elevated peak serum estradiol level of 27,000 pg/mL, which may suggest a potential cytoprotective role of elevated estrogen levels, particularly in mitigating fatal pulmonary injury-a hallmark of PQ toxicity. Following an emergency cesarean section, the neonate also survived. Crucially, toxicological analysis via liquid chromatography-tandem mass spectrometry (LC-MS/MS) detected a trace PQ concentration of 1.3 ng/mL in the neonate’s blood. This finding provides definitive quantitative evidence of transplacental transfer and effectively explains the neonate’s transient hepatic and renal dysfunction. This report underscores the importance of individualized treatment strategies, including oral ulcer management, psychological support, and monitoring of the unique physiological changes during pregnancy. While maternal dynamic plasma PQ monitoring was absent in this case, the confirmed transplacental fetal exposure highlights the critical need for comprehensive maternal-fetal toxicological assessments in future studies.

## 1 Background

Paraquat (PQ) is a highly toxic herbicide widely used in agriculture under various trade names such as Gramoxone, Tornado, and Grass Withered (Stuart et al., 2023). As a bipyridyl compound, PQ is highly valued for its potent herbicidal activity; however, its widespread application has raised significant health concerns due to its extreme toxicity to humans and animals (Donaher and Van den Hurk, 2023). Exposure can occur through dermal absorption, inhalation, or, most critically, ingestion. Even a small oral intake, typically as little as 10–15 mL of a 20%–40% solution, can lead to fatal poisoning (Yadav et al., 2023). Following ingestion, PQ undergoes rapid systemic distribution, preferentially accumulating in highly perfused organs such as the lungs, liver, kidneys, and heart, leading to severe toxicity and high mortality rates (Du et al., 2024). Fatality rates following oral ingestion range from 85% to 95%, making PQ poisoning one of the deadliest forms of toxic exposure (Deuster et al., 2023).

The primary mechanism of PQ toxicity is its ability to undergo redox cycling, generating excessive reactive oxygen species (ROS), which induce widespread oxidative damage, particularly in the pulmonary system. This oxidative stress triggers inflammation, alveolar epithelial injury, and progressive pulmonary fibrosis, ultimately leading to respiratory failure, which is the primary cause of death in severe cases (Subbiah and Tiwari, 2021). Despite ongoing advancements in toxicology and critical care medicine, no specific antidote for PQ poisoning has been identified. Current management strategies focus on limiting gastrointestinal absorption through gastric lavage and promoting systemic elimination via hemodialysis or hemoperfusion (Li et al., 2023). However, these interventions often show limited efficacy due to PQ’s rapid cellular uptake and irreversible tissue damage.

The management of PQ poisoning becomes even more complex in pregnant patients, where both maternal and fetal survival must be considered, posing significant challenges to treatment decisions (Kamath et al., 2021). Pregnancy introduces additional physiological variables that may influence the pharmacokinetics and toxicity of PQ, such as increased blood volume, altered renal clearance, and changes in immune regulation (Li et al., 2022). Furthermore, the potential transplacental transfer of PQ raises concerns regarding fetal toxicity, complicating clinical outcomes and treatment approaches (Shi et al., 2022). Given these complexities, the survival of a 32-week pregnant patient following the ingestion of a fulminant dose of PQ represents an exceedingly rare and unexpected clinical outcome. This case highlights the need for further investigation into pregnancy-associated protective factors, including estrogen, that may modulate PQ-induced toxicity.

One potential factor that may alter the clinical trajectory of PQ poisoning during pregnancy is estrogen, a hormone markedly elevated during gestation. Estrogen has been well-documented for its cytoprotective properties, particularly in attenuating oxidative stress, modulating mitochondrial function, and reducing inflammatory responses in lung tissue (Cervantes et al., 2022; Lagranha et al., 2018). Recent evidence suggests that estrogen may play a critical role in mitigating pulmonary injury and preventing the progression of oxidative damage (Hussman, 2020; Zhu et al., 2024). Therefore, we present an exceedingly rare case of a 32-week pregnant patient who survived a fulminant ingestion of PQ. This specific clinical presentation provides a unique opportunity to explore how pregnancy-associated physiological factors-particularly a peak hyper-estrogenic state-may offer critical cytoprotection against otherwise fatal PQ-induced lung injury.

## 2 Case report

### 2.1 Case presentation

A 26-year-old woman, at 32 weeks of gestation, was admitted to the Department of Occupational Disease and Poisoning Medicine, The First Affiliated Hospital of Chongqing Medical and Pharmaceutical College in May 2024 following the intentional ingestion of approximately 20–25 mL of PQ after a familial dispute. Within 2 h of ingestion, she developed nausea and vomiting, with a significant presence of green gastric contents. The patient remained conscious, with no reported seizures or loss of bladder or bowel control. Her family promptly transported her to the hospital for emergency evaluation and management.

Upon admission, her vital signs were as follows: temperature, 36.6 °C; heart rate, 102 beats/min; respiratory rate, 23 breaths/min; and blood pressure, 105/62 mmHg. Although she was alert, she exhibited signs of fatigue. Physical examination upon admission revealed severe hemorrhagic erosions and ulcerative destruction across the lips and oral mucosa (Figure 1A), and generalized shivering was observed. Scleral examination showed no icterus, and her pupils were equal, round, and reactive to light. Pulmonary auscultation revealed clear breath sounds bilaterally without adventitious sounds. Cardiac examination indicated tachycardia with a regular rhythm, and no pathological murmurs were detected. Abdominal examination showed mild distension with tenderness, but no hepatosplenomegaly was noted. Musculoskeletal examination confirmed a full range of motion in all extremities.

**FIGURE 1:**
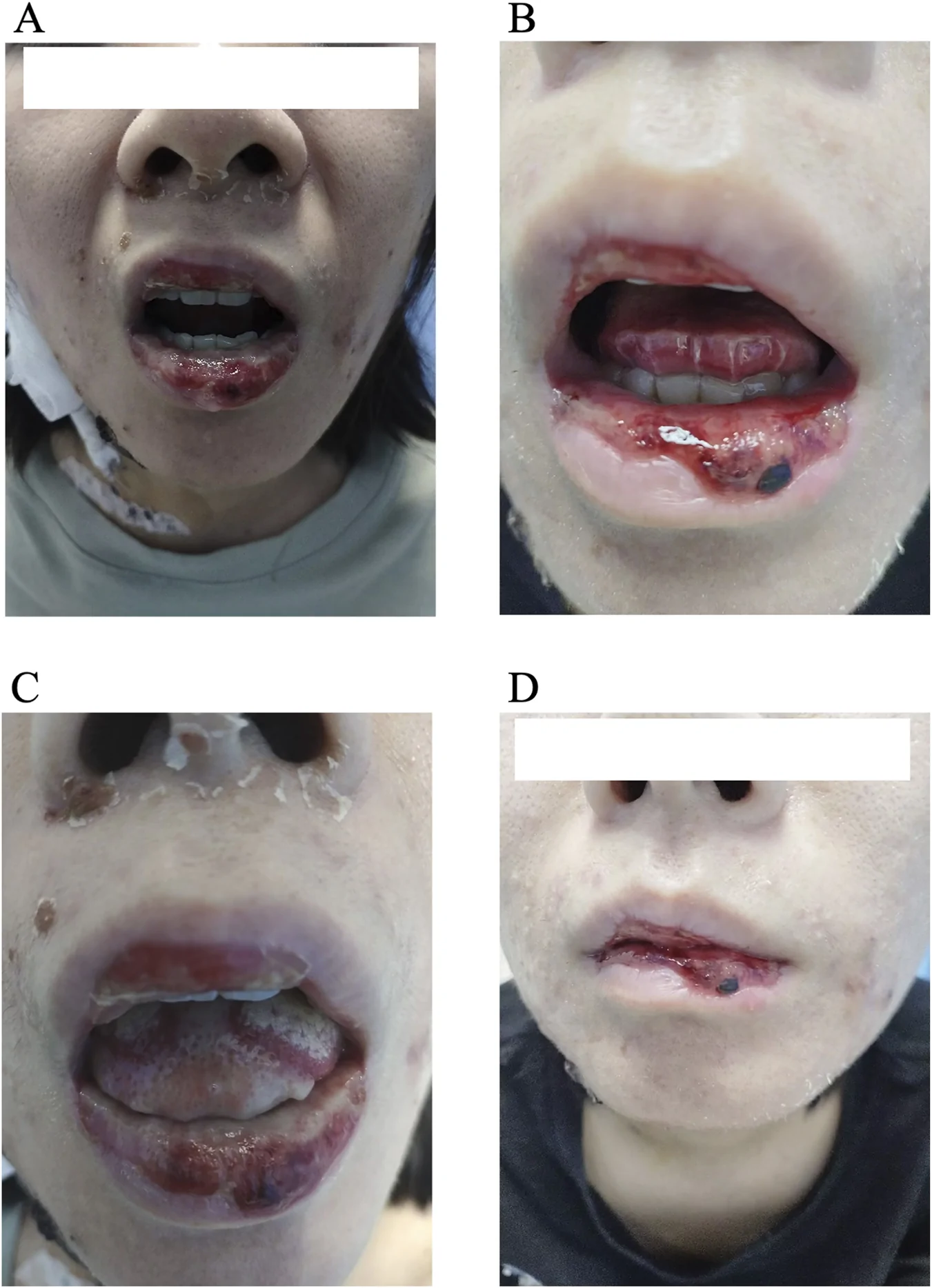
Progressive changes in oral mucosa during hospitalization and treatment in a 32-week pregnant patient with fulminant paraquat poisoning. **(A)** Initial physical examination upon admission revealed severe ulcerative destruction, crusting, and hemorrhagic erosions across both the upper and lower lips and oral mucosa. **(B)** as the condition progressed, patchy hemorrhagic filaments emerged in the oropharyngeal region, potentially indicative of vascular fragility, inflammatory damage, or early signs of coagulopathy. **(C)** following the initiation of an advanced oral mucosal care regimen-including rehabilitation mouthwash, fuzifu, saline irrigation, lidocaine, and methylcobalamin-progressive mucosal healing was observed, with reduced inflammation and enhanced epithelialization. **(D)** before discharge, the patient demonstrated significant mucosal recovery, with improved tissue integrity and minimal residual lesions. Comprehensive post-hospitalization guidance, including scheduled follow-ups and pulmonary function exercises, was provided to support continued recovery.

### 2.2 Immediate management

Initial management focused on limiting PQ absorption and promoting toxin elimination. Gastric lavage was performed with 4% sodium bicarbonate (NaHCO_3_), followed by the administration of 50 g of activated charcoal and 30 g of magnesium sulfate (MgSO_4_) for gastrointestinal decontamination. Additionally, 20% mannitol was administered to facilitate diuresis. Urinary PQ concentration was measured at 20.0 μg/mL, confirming substantial systemic exposure. Concurrently, comprehensive baseline laboratory investigations were conducted. Notably, the patient’s baseline serum estradiol (E2) level was markedly elevated at 27,000 pg/mL. Upon retrospective verification of the laboratory records, this precise value and unit were confirmed to be accurate. While exceptionally high compared to non-pregnant states, this concentration falls squarely within the established physiological reference range for the late third trimester (which can extend up to 30,000 pg/mL depending on the clinical assay), accurately reflecting a peak physiological hyper-estrogenic state.

Given the advanced gestational age and the high risk associated with maternal PQ toxicity, an urgent obstetric consultation was conducted. Ultrasonography confirmed a single viable fetus at 32 weeks, demonstrating regular cardiac activity and frequent fetal movements. Due to the anticipated severity of systemic toxicity and the risk to both maternal and fetal survival, a multidisciplinary team comprising obstetricians, intensivists, and toxicologists recommended an emergency cesarean section. Following informed consent from the family, the patient underwent surgery, and a female neonate weighing 2,600 g was successfully delivered with stable vital signs. The newborn was subsequently transferred to the neonatal intensive care unit (NICU) for close monitoring.

Postoperatively, the patient was admitted to the intensive care unit (ICU) for further management. A comprehensive therapeutic regimen was initiated, including: (1) intravenous high-dose vitamin C (3–5 g/day) to mitigate oxidative stress and promote detoxification. (2) diuretic therapy (furosemide) to enhance PQ excretion. (3) dexamethasone (30 mg/day) to reduce pulmonary inflammation and minimize fibrosis risk. (4) edaravone as an antioxidant to neutralize ROS. (5) broad-spectrum antibiotics for prophylaxis against secondary infections. (6) pantoprazole to suppress gastric acid secretion and prevent mucosal injury. (7) hemopexin therapy for organ protection. (8) hemoperfusion with a perfusion duration of 2 hours to facilitate toxin clearance. (9) uterotonics to promote uterine contraction and prevent postpartum hemorrhage. (10) psychological support to address emotional distress and mental wellbeing.

### 2.3 Clinical course and follow-up

By the third day of hospitalization, the patient remained alert and hemodynamically stable but reported mild chest tightness, dyspnea, and throat discomfort with increased pharyngeal secretions. Examination of the oral cavity revealed a white, greasy coating over the buccal mucosa, tongue, and posterior pharyngeal wall, with patchy white film that did not bleed upon contact. Additionally, the patient experienced mild discomfort at the cesarean section incision site.

#### 2.3.1 Physical examination

Respiratory rate: 25 breaths/min. Oxygen saturation: maintained within normal range on room air. Cardiac assessment: regular rhythm, no new murmurs. Pulmonary auscultation: clear breath sounds bilaterally, no adventitious sounds.

#### 2.3.2 Blood gas analysis

Blood gas and biochemical analyses on Day 3 (Table 1) revealed multiple systemic abnormalities. The patient demonstrated respiratory alkalosis with mild hypoxemia. Concurrently, biochemical markers indicated hypokalemia, early acute kidney injury (AKI), and hypoalbuminemia. Liver function parameters suggested early hepatic injury and cholestasis. Furthermore, elevated high-sensitivity troponin I and B-type natriuretic peptide (BNP) levels indicated cardiac stress and potential myocardial injury. These findings highlighted the onset of a complex pathophysiological state requiring multidisciplinary monitoring.

**TABLE 1:** Blood gas and biochemical parameters of a 32-week pregnant patient with fulminant paraquat poisoning on day 3 of hospitalization.

Biochemical test parameters	Results	Range of normal value
pH	7.45	7.35 ∼ 7.45
PCO_2_	27 mmHg ↓	35.00 ∼ 45.00
PO_2_	84 mmHg ↓	80 ∼ 100 mmHg
Potassium (K^+^)	2.9 mmol/L ↓	3.5 ∼ 4.5 mmol/L
Hematocrit (Hct)	35%	35% ∼ 45%
Serum creatinine	113.2 μmol/L ↑	41.0 ∼ 73.0 μmol/L
Urea	20.2 mmol/L ↑	2.60 ∼ 7.50 mmol/L
Albumin	32.3 g/L ↓	35 ∼ 55 g/L
Total bilirubin	72.0 μmol/L ↑	3.0 ∼ 22.0 μmol/L
Direct bilirubin	59.0 μmol/L ↑	0 ∼ 6.8 μmol/L
Gamma-glutamyl transferase (GGT)	116 U/L ↑	0 ∼ 40 U/L
Alanine aminotransferase (ALT)	93 U/L ↑	<35 U/L
High-sensitivity troponin I	0.22 μg/L ↑	0.02 ∼ 0.13 μg/L
B-type natriuretic peptide (BNP)	850 pg/mL ↑	0 ∼ 100 pg/mL

#### 2.3.3 Chest imaging results

On Day 3, the chest CT revealed a symmetric chest with increased lung markings and pleural thickening. No significant infiltrative lesions were observed (Figure 2A). These findings are consistent with the early stage of PQ poisoning, where the lung changes are primarily related to inflammatory response and initial pulmonary injury, without clear signs of consolidation or infiltrative pathology.

**FIGURE 2:**
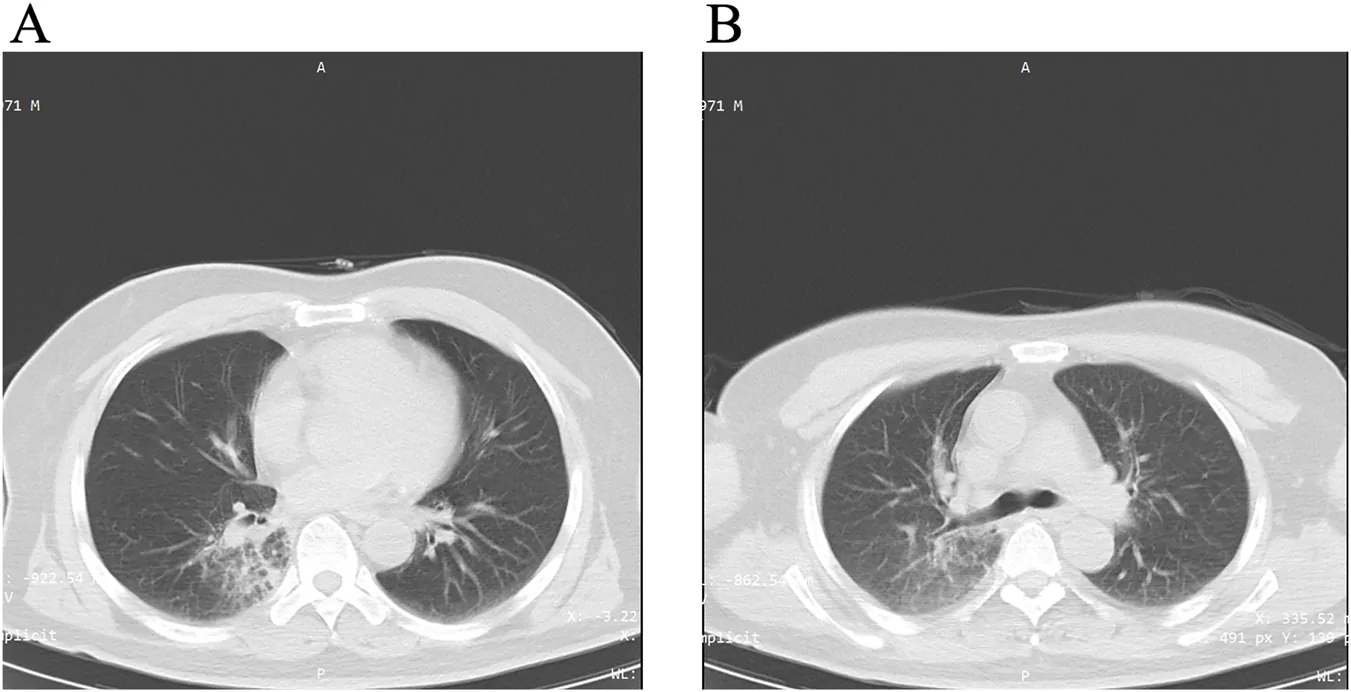
CT imaging of pulmonary changes in a 32-Week pregnant patient with fulminant paraquat poisoning. **(A)** Chest CT on Day 3: Symmetric chest with increased lung markings and pleural thickening, without significant infiltrative lesions. **(B)** Chest CT on Day 5: Symmetric chest with increased lung markings, consolidation in the right middle lobe with “air bronchogram” sign, and exudative lesions in the left lower lobe. This image corresponds to the progression of pulmonary injury during the acute phase of PQ poisoning, with early signs of respiratory failure and organ dysfunction.

#### 2.3.4 Treatment strategy

In response to the patient’s evolving clinical condition, the following therapeutic interventions were initiated: (1) hepatoprotective therapy to mitigate elevated bilirubin levels and liver enzyme abnormalities. (2) myocardial support with cyclophosphamide adenosine glucosamine to enhance cardiac function. (3) parenteral nutritional supplementation via Kavin injection to support metabolic demands. (4) blood purification therapy with hemoperfusion, conducted for a duration of 2 hours to facilitate toxin clearance.

#### 2.3.5 Oral mucosal management

Given the presence of oral mucosal erosion and the risk of secondary infection, a structured oral care regimen was implemented: (1) routine oral hygiene with sterile cotton swabs, performed three times daily (morning, midday, and evening). (2) use of soft-bristled toothbrushes to minimize mechanical trauma to the compromised mucosa. (3) mandatory oral rinsing before and after meals to reduce bacterial load. (4) antiseptic and anti-inflammatory gargle therapy, consisting of 1.0 g metronidazole and 5 mg dexamethasone diluted in 250 mL saline, administered every 5 h with a 5-min retention period per session.

### 2.4 Clinical progression and symptomatology (Day 5)

Arterial blood gas and laboratory profiles on Day 5 (Table 2) demonstrated progressive multi-organ deterioration. The patient exhibited worsening hypoxemia alongside hematological abnormalities, including thrombocytopenia and an elevated neutrophil percentage, suggesting a persistent inflammatory response. Renal and hepatic function markers indicated worsening AKI and significant hepatocellular injury with cholestasis. Cardiac biomarkers remained markedly elevated. Given these findings, multi-organ dysfunction syndrome (MODS) was strongly suspected, necessitating escalated organ support and intensive monitoring.

**TABLE 2:** Blood gas and biochemical parameters of a 32-week pregnant patient with fulminant paraquat poisoning on day 5 of hospitalization.

Biochemical test parameters	Results	Range of normal value
pH	7.38	7.35 ∼ 7.45
PCO_2_	29 mmHg ↓	35.00 ∼ 45.00
PO_2_	52 mmHg ↓	80 ∼ 100mmHg
Potassium (K^+^)	3.1 mmol/L ↓	3.5 ∼ 4.5 mmol/L
Hematocrit (Hct)	36%	35% ∼ 45%
White blood cell count	12.1 × 10^9^/L ↑	3.5 ∼ 9.5 × 10^9^/L
Platelets	63 × 10^9^/L ↓	125 ∼ 350 × 10^9^/L
Neutrophils	83.0%↑	40% ∼ 75%
Serum creatinine	133.2 μmol/L ↑	41 ∼ 73 μmol/L
Urea	25 mmol/L ↑	2.60 ∼ 7.50 mmol/L
Albumin	32.4 g/L ↓	35 ∼ 55 g/L
Total bilirubin	119.0 μmol/L ↑	3.0 ∼ 22.0 μmol/L
Direct bilirubin	106.0 μmol/L ↑	0 ∼ 6.8 μmol/L
Indirect bilirubin	22.8 μmol/L ↑	1.7 ∼ 12 μmol/L
Alanine aminotransferase (ALT)	116 U/L ↑	0 ∼ 40 U/L
Aspartate aminotransferase (AST)	78 U/L ↑	<35 U/L
GGT	127 U/L ↑	7.0 ∼ 45.0 U/L
High-sensitivity troponin I	1.06 μg/L ↑	0.02 ∼ 0.13 μg/L
BNP	1,030 pg/mL ↑	0 ∼ 100 pg/mL

#### 2.4.1 Chest CT findings

Follow-up chest CT imaging demonstrated a progressive deterioration in pulmonary status, with: (1) increased pulmonary markings. (2) consolidative lesions in the right middle lobe, exhibiting an air bronchogram pattern. (3) exudative changes in the left lower lobe, indicative of ongoing inflammatory or infectious processes (Figure 2B).

The patient was diagnosed with acute respiratory failure, accompanied by progressive hepatic, renal, and myocardial dysfunction, necessitating an escalation in therapeutic interventions.

#### 2.4.2 Revised treatment protocol

In light of the patient’s clinical deterioration, modifications were made to the treatment strategy: (1) adjustment of immunosuppressive and corticosteroid therapy to modulate systemic inflammation. (2) targeted organ support therapy, incorporating hepatoprotective and myocardial supportive agents to mitigate further deterioration. (3) continuous fluid and electrolyte management, with real-time monitoring to maintain homeostasis. (4) continuation of hemoperfusion therapy to enhance systemic toxin clearance and limit oxidative damage.

#### 2.4.3 Multidisciplinary nursing care plan

A comprehensive nursing protocol was implemented to address symptom progression and ensure optimal supportive care: (1) systematic oral ulcer assessment, employing standardized techniques to monitor lesion evolution. (2) oral cavity pH testing using test strips, guiding the selection of appropriate oral rinses to maintain mucosal integrity. (3) fungal culture of tongue erosions, with antifungal therapy initiated if laboratory findings confirmed secondary infection. (4) pain management strategies, including lidocaine hydrochloride gargle (0.1 g lidocaine in 100 mL saline) administered prior to meals, with pain levels continuously assessed and additional analgesics provided as needed. (5) anti-inflammatory gargle therapy, utilizing 10 mg dexamethasone in 250 mL saline, administered every 4 hours. (6) mucosal protection with milk gargles, aimed at forming a protective coating over the affected surfaces. (7) topical application of burn cream, using sterile swabs to promote healing and prevent secondary bacterial invasion. (8) psychological support, facilitated by consultation with neonatal medicine specialists to address postpartum anxiety and emotional distress. (9) individualized nutritional support, developed in collaboration with clinical nutrition experts to meet the metabolic demands associated with post-cesarean recovery and PQ toxicity.

### 2.5 Clinical progression and symptomatology (Day 10)

By the 10 days of hospitalization, the patient continued to experience persistent dyspnea, accompanied by progressive jaundice, which was evident in the skin, mucous membranes, and sclera, suggesting hepatic dysfunction and bilirubin accumulation. In addition, there was a marked increase in pharyngeal secretions, raising concerns about oropharyngeal inflammation and potential aspiration risk. Examination of the oral cavity revealed painful vesicular lesions on the tongue and pharyngeal mucosa, indicative of mucosal erosion and ongoing epithelial damage. Notably, patchy hemorrhagic filaments were observed in the oropharyngeal region, which may be associated with vascular fragility, inflammatory damage, or early signs of coagulopathy (Figure 1B).

Biochemical analysis on Day 10 (Table 3) confirmed persistent and worsening hepatic impairment, characterized by further elevations in bilirubin and liver enzymes. Renal function remained compromised with persistently elevated creatinine and urea. Cardiac strain continued to worsen, as evidenced by rising BNP levels. The combination of these findings confirmed the ongoing risk of MODS and underscored the critical need for intensified organ support and careful fluid management.

**TABLE 3:** Biochemical parameters of a 32-week pregnant patient with fulminant paraquat poisoning on day 10 of hospitalization.

Biochemical test parameters	Results	Range of normal value
Blood creatinine	137.2 μmol/L↑	41 ∼ 73 μmol/L
Urea	25 mmol/L↑	2.60 ∼ 7.50 mmol/L
Albumin	33.0 g/L↓	35 ∼ 55 g/L
Total bilirubin	123.0 μmol/L↑	3.0 ∼ 22.0 μmol/L
Direct bilirubin	146.0 μmol/L↑	0 ∼ 6.8 μmol/L
Indirect bilirubin	45.0 μmol/L↑	1.7 ∼ 12 μmol/L
ALT	128 U/L↑	0 ∼ 40 U/L
AST	117 U/L↑	<35 U/L
GGT	157 U/L↑	7.0 ∼ 45.0 U/L
BNP	1,172 pg/mL↑	0 ∼ 100 pg/mL

#### 2.5.1 Chest CT findings

Follow-up chest CT imaging demonstrated significant resolution of the previously noted solid lesion in the right middle lobe. However, newly developed consolidative changes were observed in the posterior segments of both lower lobes, with an increased area of involvement compared to prior imaging.

#### 2.5.2 Updated treatment protocol

In response to the evolving clinical status, the therapeutic strategy was adjusted as follows: (1) escalation of antimicrobial therapy, with a switch to meropenem (1.0 g, every 8 h, Q8H) for enhanced broad-spectrum bacterial coverage. (2) advanced oral mucosal care, incorporating a multimodal regimen comprising rehabilitation mouthwash, fuzifu, 250 mL saline, lidocaine (0.1 g), and methylcobalamin (500 μg) for symptomatic relief and mucosal healing (Figure 1C). (3) cold therapy for hemostasis, employing 0 °C–4 °C saline-soaked cotton swabs for oral cavity cleansing. Additionally, the patient performed cold saline gargling (10–15 mL per session, 2–3 min per session, 2–3 times daily) to reduce mucosal bleeding and irritation.

### 2.6 Clinical course and imaging (Day 14)

By day 14, chest CT scans demonstrated progressive resolution of the previously identified lesions, with a noticeable reduction in consolidation volume and attenuation of inflammatory changes. In addition, chest CT findings confirmed a gradual reduction in the size of pulmonary consolidations, indicating a favorable response to the ongoing treatment regimen.

### 2.7 Clinical progression (Day 18)

On the 18 days post-admission, the patient continued to experience mild exertional dyspnea, indicative of ongoing pulmonary function impairment. A structured respiratory rehabilitation program was implemented to facilitate lung function recovery and enhance ventilatory capacity.

### 2.8 Long-term recovery and discharge considerations (Day 25)

By day 25, pulmonary function remained compromised, with persistent ventilatory impairment. Follow-up chest radiography revealed residual pulmonary metaplasia, which had not been fully resorbed. Despite these findings, the patient and her family expressed a strong preference for discharge. Before discharge, the patient received comprehensive post-hospitalization guidance, including scheduled follow-up with serial chest radiographs and oral mucosal examinations in 4 weeks (Figure 1D) and pulmonary function exercises, prescribed to facilitate continued recovery of respiratory capacity.

### 2.9 Neonatal evaluation and management (Day 3 postpartum)

Three days after birth, the neonate underwent a comprehensive clinical assessment, revealing hematological abnormalities, hepatic dysfunction, and renal impairment, necessitating close monitoring and early intervention.

Hematological parameters (Table 4) suggested mild leukopenia, thrombocytopenia, and a reactive neutrophilia, indicating possible perinatal stress or systemic inflammation. Concurrently, biochemical serum chemistry results (Table 5) revealed significant neonatal hyperbilirubinemia and elevated blood creatinine, suggesting transient neonatal renal insufficiency and hepatic stress. These laboratory abnormalities directly reflected the toxicological burden on the preterm infant’s immature organ systems.

**TABLE 4:** Hematological parameters for the neonate on day 3 post-delivery following intrauterine paraquat exposure.

Biochemical test parameters	Results	Range of normal value
Leukocytes	14.13 × 10^9^/L↓	15 ∼ 20 × 10^9^/L
Platelets	131 × 10^9^/L↓	183 ∼ 614 × 10^9^/L
Neutrophils	77.60%↑	7% ∼ 56%
Hemoglobin	201 g/L↑	180 ∼ 190 g/L

**TABLE 5:** Biochemical parameters for the neonate on day 3 post-delivery following intrauterine paraquat exposure.

Biochemical test parameters	Results	Range of normal value
Blood creatinine	111.2 μmol/L↑	13 ∼ 33 μmol/L
ALT	12.2 U/L	8 ∼ 71 U/L
Total bilirubin	120.1 μmol/L↑	0 ∼ 23.0 μmol/L
Direct bilirubin	13.0 μmol/L↑	0 ∼ 7.0 μmol/L
Indirect bilirubin	107.0 μmol/L↑	0 ∼ 20 μmol/L
GGT	78.2 U/L	9.0 ∼ 150.0 U/L

#### 2.9.1 Chest CT findings

Neonatal chest CT imaging revealed: (1) multiple exudative lesions scattered throughout both lungs. (2) areas of ventilation-perfusion mismatch, suggestive of compromised gas exchange. (3) bilateral pleural effusion, raising concerns for neonatal respiratory distress syndrome (NRDS) or intrauterine inflammatory exposure. (4) CT revealed multiple exudative lesions and areas of poor ventilation in both lungs, along with bilateral pleural effusion. Chest X-ray image further supported these findings, showing bilateral pulmonary changes, including increased and blurred lung markings with patchy exudative lesions in both lower lungs. These radiological signs also indicated NRDS or intrauterine inflammatory exposure (Figure 3B).

**FIGURE 3:**
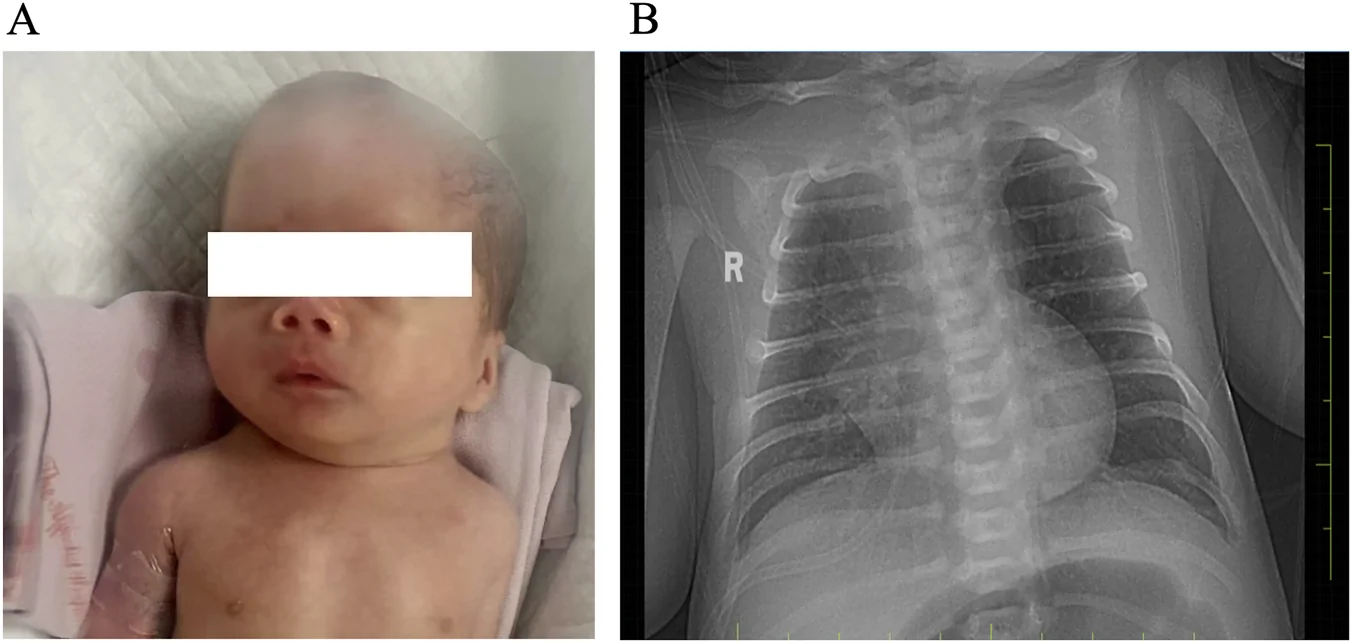
Post-treatment status of a neonate following comprehensive respiratory and supportive management after intrauterine paraquat exposure. **(A)** clinical image of the neonate at discharge following 18 days of hospitalization, demonstrating stable vital signs and improved overall condition. A multidisciplinary treatment strategy, including empirical antibiotic therapy, nebulized respiratory therapy, and continuous supportive care, was implemented. The neonate showed stabilized respiratory function, improved weight gain, and met all discharge criteria, with recommendations for continued pediatric follow-up and respiratory monitoring post-discharge. **(B)** chest X-ray image showing bilateral pulmonary changes, including increased and blurred lung markings with patchy exudative lesions in both lower lungs. These findings are suggestive of NRDS or intrauterine inflammatory exposure.

#### 2.9.2 Neonatal treatment plan

A multidisciplinary neonatal care strategy was implemented, including: (1) empirical antibiotic therapy, with cefotaxime (50 mg, every 12 h, Q12H) for broad-spectrum infection control. (2) nebulized respiratory therapy, incorporating: interferon (15 μg) for antiviral and immune-modulatory effects. Acetylcysteine (0.15 g) to facilitate mucolysis and airway clearance. 0.9% saline (3 mL) as a diluent to ensure optimal aerosolization and drug deposition. (3) continuous supportive care, including oxygen supplementation and thermoregulation, as needed, to optimize pulmonary adaptation postnatally.

After 18 days of neonatal hospitalization, the infant demonstrated stable respiratory function and adequate weight gain, meeting discharge criteria. The neonate was discharged in good health (Figure 3A), with recommendations for routine pediatric follow-up and continued respiratory monitoring post-discharge.

## 3 Discussion

Paraquat (PQ) poisoning remains a severe toxicological emergency with high morbidity and mortality (Tambuzzi et al., 2024; Cestonaro et al., 2022). In this case, the mother’s unexpected survival despite ingesting a fulminant dose (20–25 mL) is highly unusual. Typically, this ingested dose leads to rapid multi-organ failure (Tang et al., 2024; Kavianinia et al., 2025); however, the clinical course was inconsistent with this classification, as the patient did not present with typical signs of cardiogenic shock or severe arrhythmias (Yi et al., 2024). Furthermore, this case presents an unusual trajectory of pulmonary repair. While initial CT imaging showed consolidation, the lesions demonstrated significant and rapid resolution by Day 14. Considering that standard therapies typically have limited efficacy in halting or reversing progressive fibrosis once initiated, this non-traditional rapid pulmonary repair kinetics supports the presence of a distinct endogenous buffer (Gao et al., 2021; Eddleston, 2022; Tao et al., 2021).

### 3.1 The estrogenic protective hypothesis and pulmonary recovery

We hypothesize that pregnancy-related physiological changes, especially a state of physiological hyper-estrogenism (baseline serum estradiol of 27,000 pg/mL), played a central cytoprotective role in mitigating the fatal pulmonary outcomes (Peng et al., 2023). Estrogen is well-documented to attenuate oxidative stress, modulate mitochondrial function, and suppress fibroblast activation. Rather than acting in isolation, this ultra-high estradiol concentration likely served as a foundational antioxidant synergist. By pre-conditioning cellular defense mechanisms, estrogen may have significantly enhanced the efficacy of conventional free-radical scavengers (e.g., Vitamin C and Edaravone) to halt early-stage fibrosis (Lv et al., 2021; Valotto Neto et al., 2024). Additionally, pregnancy-related physiological adaptations, such as increased blood volume, enhanced renal clearance, and altered volume of distribution, might have altered the pharmacokinetics of the toxin, further buffering its toxic effects on the lungs (Li et al., 2021; Sule et al., 2022; Mohamad et al., 2020).

### 3.2 Transplacental PQ transfer and neonatal toxicity

A pivotal finding in this case is the definitive quantitative confirmation of fetal PQ exposure. Toxicological analysis of the neonate’s blood via LC-MS/MS detected a trace PQ concentration of 1.3 ng/mL (Yang et al., 2025; Li et al., 2020; Kumar et al., 2021). This provides direct evidence that PQ crosses the placental barrier. Furthermore, the stark concentration gradient-from a fulminant maternal ingestion to a mere 1.3 ng/mL fetal exposure-highlights the robust physical interception of the placental barrier alongside maternal volume distribution buffering. Importantly, although 1.3 ng/mL represents a trace amount, it effectively correlates with the transient hepatic and renal dysfunction observed in the neonate (Eke et al., 2023). The immature detoxification enzyme systems (e.g., glutathione peroxidase) and reduced renal clearance capacity of a 32-week preterm infant render them highly susceptible to oxidative stress induced by even minimal PQ accumulation (Barma et al., 2025).

### 3.3 Limitations and future perspectives

Several critical limitations must be acknowledged in this case report. First, the prompt early cesarean section removed the intense physiological burden of pregnancy, and aggressive multifactorial clinical interventions undoubtedly contributed to the favorable outcome (Dobravc Verbič et al., 2024). As an isolated case report, causal conclusions cannot be definitively drawn regarding estrogen without a non-pregnant control group (Trakulsrichai et al., 2019; See et al., 2022). Second, the absence of dynamic plasma PQ concentrations and the lack of a Severity Index of Paraquat Poisoning (SIPP) score hindered our ability to assess the severity of poisoning quantitatively and comprehensively understand the toxin’s kinetics (Lin et al., 2011; C et al., 2020). Third, the lack of serial high-resolution CT imaging between Days 5 and 14 leaves the exact chronological progression and resolution kinetics of the pulmonary injury uncertain. Finally, the neonatal outcomes are limited to the immediate post-natal period, leaving a critical gap in understanding the long-term developmental risks following confirmed intrauterine exposure (Pinheiro and Stika, 2020; Li et al., 2026).

Moving forward, future research should incorporate serial estrogen measurements and dynamic plasma PQ tracking (utilizing tools like the SIPP score) throughout the clinical course, ideally comparing pregnant cohorts with non-pregnant controls to quantify the precise magnitude of hormone-mediated protection (Kumar et al., 2021; Eke et al., 2023; Barma et al., 2025). Future studies should also aim to provide serial high-resolution CT imaging over the first 3–4 weeks to map PQ-induced lung injury dynamics during pregnancy (Dobravc Verbič et al., 2024). Furthermore, prioritizing toxicological analysis of umbilical cord and neonatal blood, alongside long-term pediatric follow-up assessments of growth, neurodevelopment, and pulmonary function, will be crucial to fully elucidating fetal toxicokinetics and the long-term impact of maternal PQ exposure on fetal health (Puris et al., 2025; Gawarammana and Buckley, 2011).

## 4 Conclusion

This report documents an exceptionally rare instance of maternal and neonatal survival following a fulminant PQ ingestion. The favorable maternal outcome, characterized by the unexpected rapid resolution of pulmonary lesions, suggests a potential cytoprotective and antioxidative buffering effect of physiological hyper-estrogenism during late pregnancy. However, it is crucial to emphasize that this association remains an observational hypothesis. A single case report cannot establish a direct, causal relationship between high estradiol levels and protection against PQ toxicity. Verifying this potential protective mechanism requires definitive proof through future original research, systematic reviews, targeted animal studies, or larger patient registries. Furthermore, the trace detection of PQ in the neonate provides critical quantitative evidence of transplacental toxicokinetics, directly correlating with transient neonatal organ dysfunction. For emergency poison management, this case underscores that an aggressive, multidisciplinary approach-integrating early hemoperfusion, advanced mucosal care, and timely obstetric intervention-remains paramount. While this case inspires novel, hormone-aware perspectives, physiological gestational buffers must be interpreted cautiously when evaluating overall prognosis.

## Data Availability

The original contributions presented in the study are included in the article/supplementary material, further inquiries can be directed to the corresponding authors.
